# Association between *MIR499A* rs3746444 polymorphism and breast cancer susceptibility: a meta-analysis

**DOI:** 10.1038/s41598-020-60442-3

**Published:** 2020-02-26

**Authors:** Shing Cheng Tan, Poh Ying Lim, Jie Fang, Mira Farzana Mohamad Mokhtar, Ezanee Azlina Mohamad Hanif, Rahman Jamal

**Affiliations:** 1 UKM Medical Molecular Biology Institute, Universiti Kebangsaan Malaysia, Kuala Lumpur, Malaysia; 2 Department of Community Health, Faculty of Medicine & Health Sciences, Universiti Putra Malaysia, Selangor, Malaysia; 3 Department of Language and Literacy Education, Faculty of Education, University of Malaya, Kuala Lumpur, Malaysia

**Keywords:** Genetic association study, Genetic markers, Cancer, Epidemiology, Genetics research

## Abstract

Numerous studies have investigated the association of *MIR499A* rs3746444 polymorphism with breast cancer susceptibility, but the results have been inconsistent. In this work, we performed a meta-analysis to obtain a more reliable estimate of the association between the polymorphism and susceptibility to breast cancer. A comprehensive literature search was conducted on PubMed, Scopus, Web of Science (WoS), China National Knowledge Infrastructure (CNKI), VIP and Wanfang databases up to January 2020. A total of 14 studies involving 6,797 cases and 8,534 controls were included for analysis under five genetic models: homozygous (GG vs. AA), heterozygous (AG vs. AA), dominant (AG + GG vs. AA), recessive (GG vs. AA + AG) and allele (G vs. A). A statistically significant association was observed between the polymorphism and an increased breast cancer susceptibility under all genetic models (homozygous, OR = 1.33, 95% CI = 1.03–1.71, P = 0.03; heterozygous, OR = 1.08, 95% CI = 1.00–1.16, P = 0.04; dominant, OR = 1.15, 95% CI = 1.02–1.30; P = 0.03; recessive, OR = 1.35, 95% CI = 1.06–1.72, P = 0.01; allele, OR = 1.12, 95% CI = 1.00–1.26, P = 0.04). Subgroup analysis based on ethnicity suggested that significant association was present only among Asians, but not Caucasians. In conclusion, *MIR499A* rs3746444 polymorphism was significantly associated with breast cancer susceptibility among Asians, suggesting its potential use as a genetic risk marker in this population.

## Introduction

Breast cancer is the most common type of cancer and the leading contributor to cancer-related deaths in women worldwide^1^. Early identification of individuals at risk of the cancer is the key to its prevention. Currently, genetic testing has emerged as a promising strategy for predicting breast cancer risk. The most regularly tested genes in breast cancer are *BRCA1* and *BRCA2*, whose mutations have been unequivocally linked to a substantially elevated risk of the cancer. However, the overall prevalence of these high penetrance mutations is low, ranging from 0.2–0.3% in the general population to approximately 20% in breast cancer patients with a family history of the malignancy^2^. Thus, mutations in *BRCA* genes have a relatively low population attributable risk (PAR) and as such, the benefit of screening for these mutations at a population-wide scale is largely debatable^3^.

Over the past decades, the potential of genetic polymorphisms as markers for breast cancer risk assessment has become increasingly apparent^4^. In contrast to high penetrance mutations, genetic polymorphisms are very common in the general population, but are typically associated with a modest risk of cancer. However, it is believed that when a large number of such polymorphisms are combined, the magnitude of the risk conferred could be very significant^5^. In recent years, polymorphisms in microRNA genes have been widely investigated as the gene products play important roles in regulating the expression of many cancer-related genes^6–8^. Among the microRNA genes that have been frequently investigated is *MIR499A*, which encodes microRNA-499a. The functions and mechanism of miR-499a in breast cancer is not well understood, but it is known that miR-499a possesses both oncogenic and tumor suppressive functions in other cancer types. For example, in colorectal cancer, miR-499a could promote invasiveness and metastatic capabilities by targeting *FOXO4* and *PDCD4* tumor suppressor genes^9^. Similarly, in hepatocellular carcinoma (HCC), the microRNA was found to target *MAPK6* to induce proliferation and migration of the cells^10,11^. However, in HCC, the microRNA was also observed to target the *ETS1* oncogene to inhibit the cancer progression^12^. Additionally, in non-small cell lung cancer, miR-499a was found to exert its tumor suppressive function by targeting *VAV3* oncogene^13^.

The mechanism of microRNA target recognition and selection is determined, at least in part, by its seed sequences, i.e. a conserved region essential for its binding to target mRNAs^14^. Thus, polymorphisms occurring in the seed region of a microRNA gene may contribute to its oncogenic or tumor suppressive functions and subsequently affect cancer risk or susceptibility. One such polymorphism is rs3746444, which results from an A-to-G substitution in the seed region of *MIR499A*. A number of studies have examined the association of the polymorphism with breast cancer susceptibility, but the results obtained have been inconsistent. For instance, while He *et al*.^15^ demonstrated that the variant allele of *MIR499A* rs3746444 polymorphism was significantly associated with an increased susceptibility to breast cancer, Morales *et al*.^16^ did not find any significant association. One of the major reasons for the inconsistency of study results in genetic association studies is the limited sample size and thus, low statistical power of the individual studies^17,18^. Therefore, in this work, we aimed to pool the study findings via a meta-analysis in order to derive a more precise estimate of the association between *MIR499A* rs3746444 polymorphism and breast cancer susceptibility.

## Methodology

### Literature search

A comprehensive literature search was performed on PubMed, Scopus, Web of Science (WoS), China National Knowledge Infrastructure (CNKI), VIP and Wanfang databases up to January 2020. No language restriction was imposed. The PubMed search terms used were as follows: [(breast neoplasms OR breast cancer) AND (MIR499A OR MIR-499 OR MIR-499A OR microRNA-499 OR microRNA-499A OR rs3746444) AND (polymorphism OR mutation OR variant OR variation)]. The search terms were modified appropriately for other databases.

Eligible studies were selected based on the following criteria: (i) those that investigated the association between *MIR499A* rs3746444 polymorphism and breast cancer susceptibility; (ii) case-control (including nested case-control) or cohort in design; and (iii) contained data on genotype and allele frequencies or sufficient data to derive this information. Non-human studies and duplicated reports were excluded. When overlapping data were found in multiple studies, only the most recent report or the one with the largest sample size was included.

### Data extraction and quality assessment

Two investigators independently extracted the following information from eligible studies: First author, year of publication, country, number of cases and controls, genotype and allele frequencies, ethnicity of the subjects (Asian vs. Caucasian), and deviation of control genotype distribution from the Hardy-Weinberg equilibrium (HWE). If data on HWE was not reported, a goodness-of-fit test was used to determine whether the observed genotype frequencies conformed to the expected distribution. The quality of the eligible studies was assessed by using the Modified Newcastle-Ottawa Scale for Case-Control Studies of Genetic Association^19^. Studies with ≥5 stars were considered to be of high quality.

### Statistical analysis

Quantitative synthesis of the data was performed by using Review Manager (version: 5.3.5), with the common allele/genotype used as the reference. The association between *MIR499A* rs3746444 polymorphism and breast cancer susceptibility was evaluated under five genetic models: homozygous (GG vs. AA), heterozygous (AG vs. AA), dominant (AG + GG vs. AA), recessive (GG vs. AA + AG) and allele (G vs. A) comparison models. Heterogeneity among the studies in each genetic model was assessed by using Cochran’s Q and I2 statistics. A P value of <0.1 and I2 value of >50% was interpreted as having significant heterogeneity. A fixed-effect method was used to calculate the pooled odds ratio (OR) and the corresponding 95% confidence interval (CI) for genetic models which did not show significant heterogeneity; otherwise a random-effect method was used. The significance of the genetic association was measured using Z test. A forest plot was constructed to graphically display the results. A subgroup analysis was performed based on ethnicity of the subjects (Asian vs. Caucasian) and methodology quality of the studies (high quality vs. low quality). Sensitivity analysis was performed by iteratively omitting one study at a time to determine the stability and robustness of the results. Publication bias was assessed by performing Begg’s test and Egger’s test using JASP (version 0.9.2.0), and by visually inspecting the funnel plot for asymmetry. For all analyses, statistically significance was assumed at P < 0.05, unless otherwise stated.

### *In silico* analysis

Prediction of miR-499a target genes was performed using DIANA Tarbase version 7.0 (http://diana.imis.athena-innovation.gr/DianaTools/index.php?r=tarbase/index) and DIANA microT-CDS (http://diana.imis.athena-innovation.gr/DianaTools/index.php?r=microT_CDS/index) tools which have been integrated with miRBase (ver. 18) and Ensembl (ver. 69). Functional annotation of the predicted target genes as well as KEGG pathway enrichment analysis was then performed by using DIANA-miRPath version 3.0 (http://snf-515788.vm.okeanos.grnet.gr/)^20–23^. The DIANA-microT threshold used was 0.8 at a P value of 0.05. Fisher’s exact test (hypergeometric statistics) and false discovery rate (FDR) correction was used for the enrichment analysis. miRmut2Go (http://compbio.uthsc.edu/miR2GO/mir2goSNP.php) was used to compare the functional similarity between the wild type and variant alleles of the *MIR499A* rs3746444 polymorphism. Furthermore, RNAfold WebServer (http://rna.tbi.univie.ac.at/cgi-bin/RNAWebSuite/RNAfold.cgi) was used to predict, based on minimum free energy (MFE) calculation, the secondary structures formed as a result of the A-to-G substitution. PolymiRTS Database 3.0 (http://compbio.uthsc.edu/miRSNP/) was used to predict whether the polymorphism affects its target recognition.

## Results

### Characteristics of the studies

**Figure 1:**
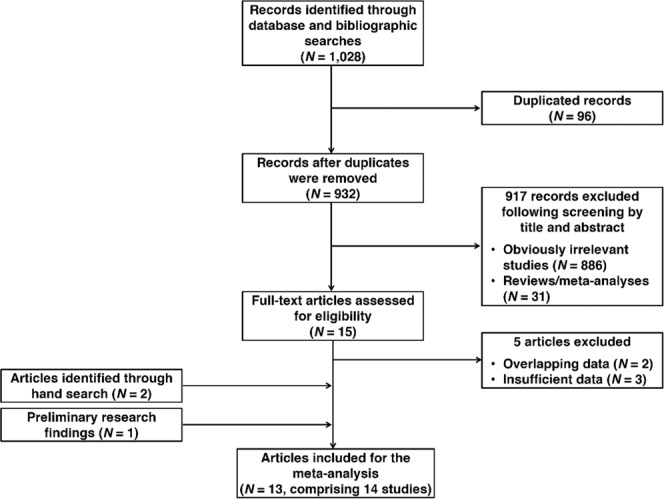
Flow diagram of study selection.

The initial search strategy resulted in the identification of 1028 records (PubMed, N = 25; Scopus, N = 931; WoS, N = 59; CNKI, N = 7; VIP, N = 1; Wanfang, N = 5). After deduplication, 932 unique records were screened by title and abstract for relevance. A total of 15 records were identified as being potentially relevant; thus, full-text articles of 16 potentially relevant records were retrieved and checked for eligibility. The reference list of these records were also hand-searched to identify additional studies. Ultimately, 12 records (which comprised 13 studies) were included in the meta-analysis^15,16,24–33^. We also included preliminary data from our laboratory in the meta-analysis, making the total number of included studies 14. The search selection process is illustrated in Fig. 1.

These 14 studies involved a total of 6,797 cases and 8,534 controls. Detailed characteristics of the included studies are shown in Table 1. Subjects in 10 of the studies belonged to Asian ethnicity, three other studies were conducted on Caucasians, while those in the remaining one study were Africans. Eight of the studies exhibited high methodological quality based on the Modified Newcastle-Ottawa Scale for Case-Control Studies of Genetic Association. The star ratings of the included studies are shown in Table 2.

**Table 1:** Characteristics of the included studies.

No.	Author	Year	Ethnicity	Population	No. of subjects (case/control)	HWE *P* value in controls
1.	Hu *et al*.^24^	2009	Asian	China	1009/1093	0.057
2.	Catucci *et al*. (a)^25^	2010	Caucasian	Italy	756/1242	0.250
3.	Catucci *et al*. (b)^25^	2010	Caucasian	Germany	823/925	0.893
4.	Alshatwi *et al*.^26^	2012	Asian	Saudi Arabia	100/100	0.227
5.	Bansal *et al*.^27^	2014	Asian	India	121/164	0.002
6.	Omrani *et al*.^28^	2014	Asian	Iran	236/203	<0.001
7.	Qi *et al*.^29^	2015	Asian	China	321/290	0.053
8.	He *et al*.^15^	2015	Asian	China	450/450	0.143
9.	Dai *et al*.^30^	2015	Asian	China	560/583	0.131
10.	Qian *et al*.^32^	2016	African	Multiple	1657/2028	0.288
11.	Afsharzadeh *et al*.^33^	2017	Asian	Iran	100/150	0.633
12.	Morales *et al*.^16^	2018	Caucasian	Chile	440/1048	0.836
13.	Doulah *et al*.^31^	2018	Asian	Iran	80/100	0.901
14.	Tan *et al*.	Unpublished	Asian	Malaysian	144/158	0.165

**Table 2:** Assessment of the quality of the included studies.

Study	Selection				Comparability	Exposure			Total star
	Criteria 1	Criteria 2	Criteria 3	Criteria 4	Criteria 1	Criteria 1	Criteria 2	Criteria 3	
Hu *et al*.^24^	★	★	★	★	★	★	★		7
Catucci *et al*. (a)^25^	★	★	★	★	★		★		6
Catucci *et al*. (b)^25^	★	★	★	★	★		★		6
Alshatwi *et al*.^26^	★	★		★			★		4
Bansal *et al*.^27^	★	★		★	★		★		5
Omrani *et al*.^28^	★	★		★			★		4
Qi *et al*.^29^		★	★	★					3
He *et al*.^15^	★	★	★	★			★		5
Dai *et al*.^30^	★	★	★	★	★		★		6
Qian *et al*.^32^	★	★	★	★	★★		★		7
Afsharzadeh *et al*.^33^		★		★			★		3
Morales *et al*.^16^	★	★	★	★	★★	★	★		8
Doulah *et al*.^31^		★		★			★		3
Tan *et al*.	★			★		★	★		4

### Quantitative synthesis

**Figure 2:**
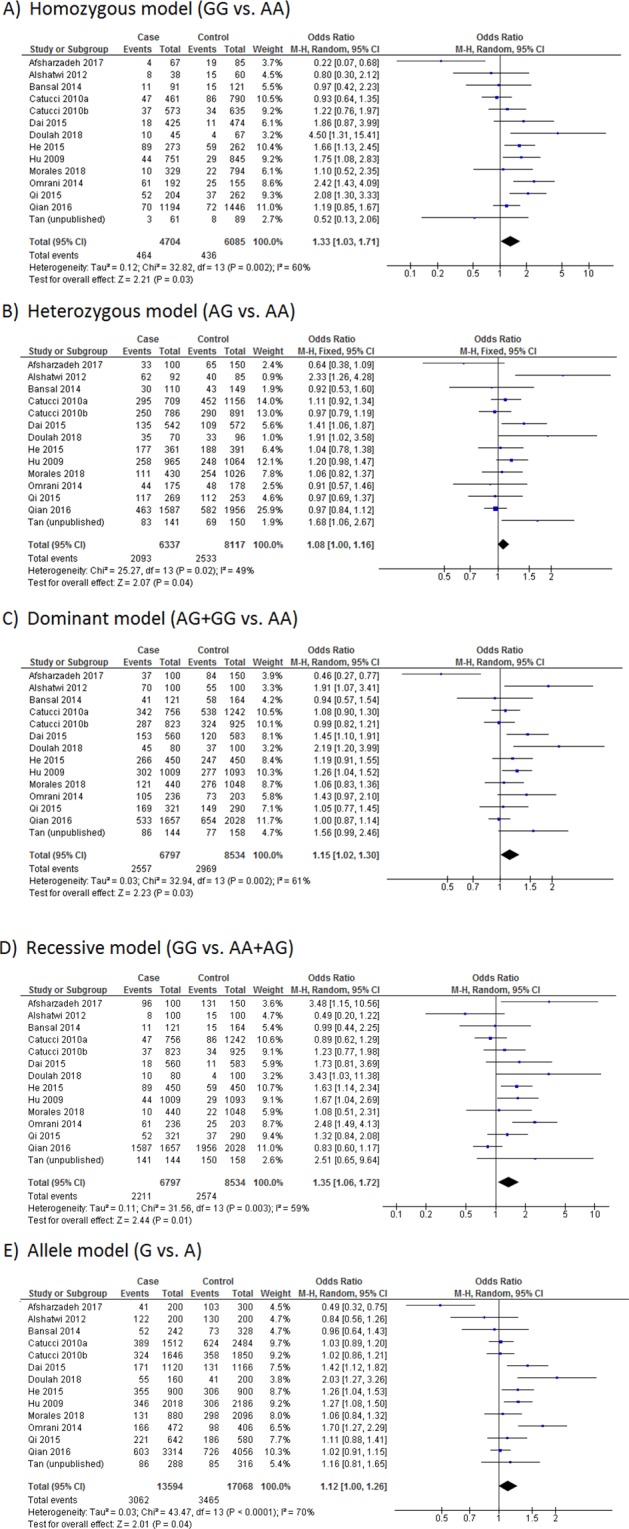
Forest plots of the association between *MIR499A* rs3746444 polymorphism and breast cancer susceptibility.

The pooled association of *MIR499A* rs3746444 polymorphism with breast cancer susceptibility is summarized in Table 3. Overall, the polymorphism was found to be significantly associated with breast cancer susceptibility in all genetic models (homozygous model, OR = 1.33, 95% CI = 1.03–1.71, P = 0.03; heterozygous model, OR = 1.09, 95% CI = 1.01–1.17, P = 0.02; dominant model, OR = 1.15, 95% CI = 1.02–1.30, P = 0.03; recessive model, OR = 1.35, 95% CI = 1.06–1.72, P = 0.01; allele model, OR = 1.12, 95% CI = 1.00–1.26, P = 0.04) (Fig. 2). Subgroup analysis by ethnicity revealed that the statistically significant positive association was present only among Asians (P < 0.05 in all genetic models except allele model), but not among Caucasians (P > 0.05) (Table 3). In the allele model, a borderline lack of significance was observed (P = 0.08). In addition, when stratified by methodology quality of the studies, high quality studies exhibited a significant (or borderline lack of significant) positive association in all genetic models, whereas low quality studies showed a significant association only in recessive model (Table 3).

**Table 3:** Summary of the association between *MIR499A* rs3746444 polymorphism and breast cancer susceptibility.

Comparison model	No. of studies	No. of cases	No. of controls	Effect model	OR (95% CI)	P
**Homozygous model**						
Overall	14	4,704	6,085	Random	1.33 (1.03–1.71)	0.03
Asian	10	2,147	2,420	Random	1.45 (1.01–2.07)	0.04
Caucasian	3	1,363	2,219	Fixed	1.04 (0.79–1.37)	0.79
High quality	8	4,097	5,367	Fixed	1.28 (1.09–1.51)	<0.01
Low quality	6	607	718	Random	1.23 (0.60–2.53)	0.57
**Heterozygous model**						
Overall	14	6,337	8,117	Fixed	1.08 (1.00–1.16)	0.04
Asian	10	2,825	3,088	Fixed	1.18 (1.05–1.32)	<0.01
Caucasian	3	1,925	3,073	Fixed	1.04 (0.92–1.18)	0.48
High quality	8	5,490	7,205	Fixed	1.07 (0.99–1.15)	0.11
Low quality	6	847	912	Random	1.24 (0.86–1.79)	0.26
**Dominant model**						
Overall	14	6,797	8,534	Random	1.15 (1.02–1.30)	0.03
Asian	10	3,121	3,291	Random	1.24 (1.02–1.50)	0.03
Caucasian	3	2,019	3,215	Fixed	1.04 (0.93–1.17)	0.47
High quality	8	5,816	7,533	Fixed	1.09 (1.02–1.18)	0.02
Low quality	6	981	1,001	Random	1.27 (0.86–1.88)	0.23
**Recessive model**						
Overall	14	6,797	8,534	Random	1.35 (1.06–1.72)	0.01
Asian	10	3,021	3,141	Fixed	1.60 (1.32–1.93)	<0.01
Caucasian	3	2,019	3,215	Fixed	1.02 (0.77–1.33)	0.91
High quality	8	5,816	7,533	Fixed	1.16 (0.99–1.37)	0.07
Low quality	6	981	1,001	Random	1.78 (1.03–3.08)	0.04
**Allele model**						
Overall	14	13,594	17,068	Random	1.12 (1.00–1.26)	0.04
Asian	10	6,242	6,582	Random	1.17 (0.98–1.40)	0.08
Caucasian	3	4,038	6,430	Fixed	1.03 (0.94–1.14)	0.52
High quality	8	11,632	15,066	Fixed	1.11 (1.04–1.17)	<0.01
Low quality	6	1,962	2,002	Random	1.11 (0.78–1.58)	0.56

### Sensitivity analysis

For heterozygous and recessive models, the results remained largely unchanged when any of the studies was removed from the analysis. However, in other models, the results were less stable. In particular, the statistical significance of homozygous, dominant and allele models diminished when data from Dai *et al*.^30^ was omitted. A similar observation was documented in homozygous and allele models when data from Hu *et al*.^24^ was removed. In addition, the homozygous model was also heavily influenced by Qi *et al*.^29^, while the allele model was heavily influenced by Doulah *et al*.^31^, He *et al*.^15^, Omrani *et al*.^28^ and Qi *et al*.^29^ Despite this, the direction and magnitude of the association were not affected by any of the studies, and the P values were still at the borderline of statistical significance.

### Publication bias

**Figure 3:**
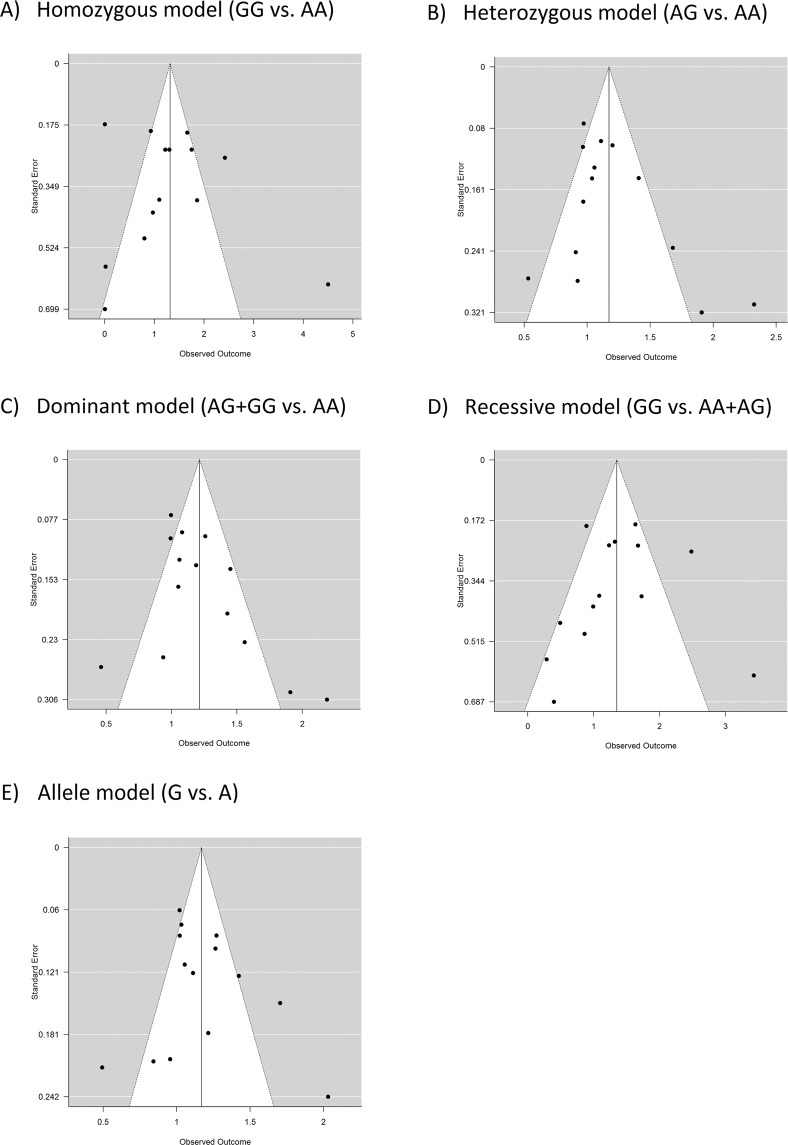
Funnel plots for assessing publication bias.

Funnel plots for publication bias are shown in Fig. 3. Visual inspection of the funnel plots did not reveal any evidence of publication bias. Formal tests using Begg’s and Egger’s methods also did not find any significant publication bias (homozygous model, Begg’s test P = 0.667, Egger’s test P = 0.787; heterozygous model, Begg’s test P = 0.388, Egger’s test P = 0.089; dominant model, Begg’s test P = 0.062, Egger’s test P = 0.055; recessive model, Begg’s test P = 0.279, Egger’s test P = 0.518; allele model, Begg’s test P = 0.518, Egger’s test P = 0.706).

### *In silico* analysis

**Figure 4:**
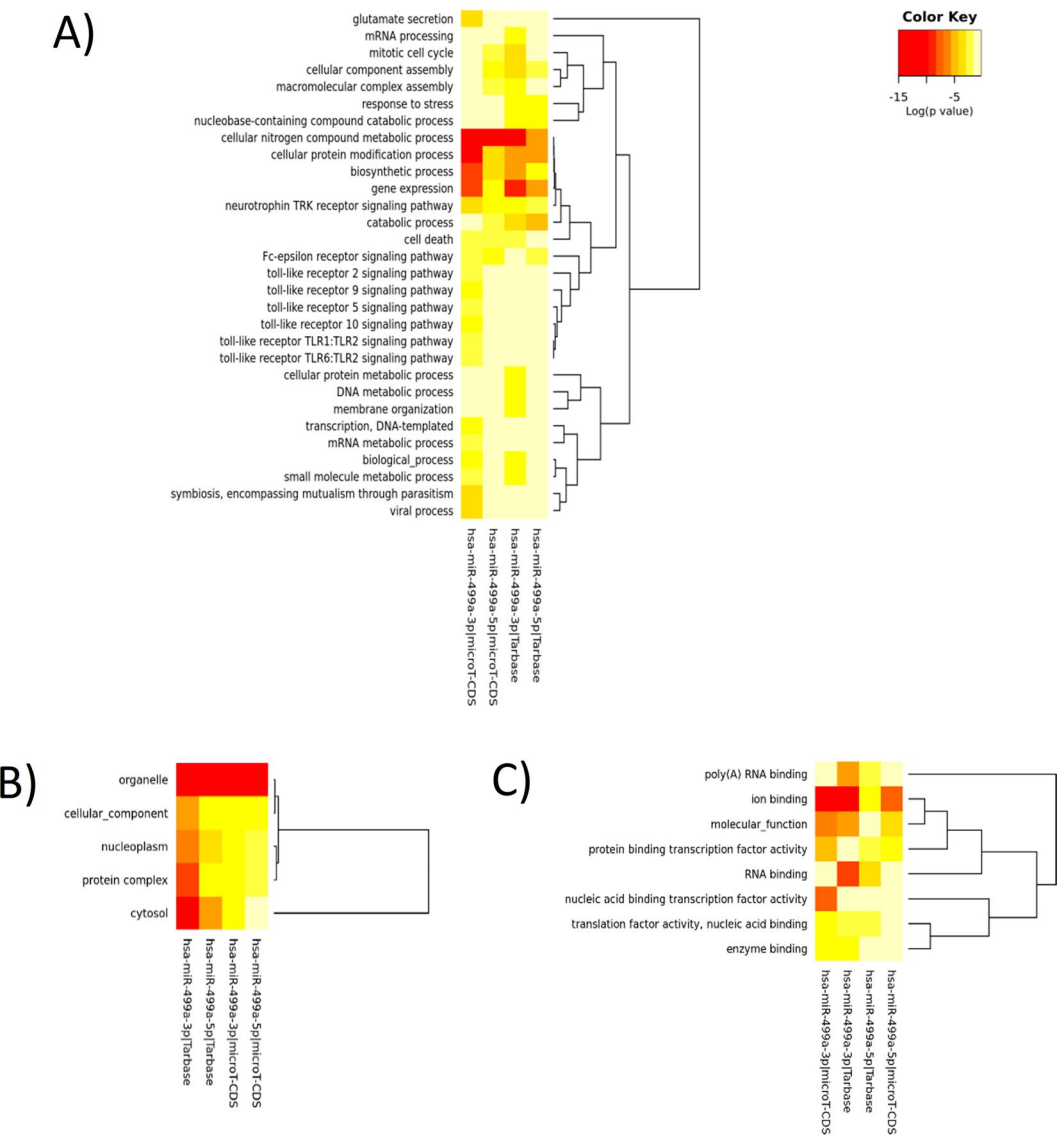
Heat map showing (**A**) biological processes (**B**) cellular components and (**C**) molecular functions of predicted miR-499a target genes.

The DIANA Tarbase v7.0 and DIANA microT-CDS identified a total of 2,286 target genes for miR-499a. Of these, 1,169 were targeted by the 3p arm, 899 by the 5p arm, and 218 by both arms of miR-499a. Pathway enrichment analysis of these target genes revealed that transcriptional misregulation in cancer is the most common pathway involved (28 genes), followed by RNA transport (26 genes), ubiquitin mediated proteolysis (24 genes), cell cycle and hippo signaling pathway (22 genes) (Table 4). Functional annotation of target genes based on the biological processes, cellular components and molecular functions is shown in Fig. 4. It was noted that the most prominent biological process involved is the Toll-like receptor signaling pathway.

**Table 4:** DIANA miRPath KEGG pathway enrichment analysis of the miR-499a target genes.

KEGG pathway	KEGG pathway ID	p-value	Found genes	miRNAs
Transcriptional misregulation in cancer	hsa05202	2.15E-05	28	4
Biotin metabolism	hsa00780	3.28E-05	1	1
Thyroid hormone signaling pathway	hsa04919	0.000244899	19	4
Cell cycle	hsa04110	0.000695201	22	4
Sulfur relay system	hsa04122	0.003319243	2	2
Hippo signaling pathway	hsa04390	0.005447739	22	4
RNA transport	hsa03013	0.009415218	26	4
Ubiquitin mediated proteolysis	hsa04120	0.021323824	24	4
Hedgehog signaling pathway	hsa04340	0.027238434	11	3
Prostate cancer	hsa05215	0.029004416	17	4

Analysis with PolymiRTS v3.0 showed that the A-to-G substitution in the rs3746444 polymorphism created new target sites for 763 genes and disrupted the target sites of 2,408 genes. Besides, miRmut2Go revealed that the A and G alleles had low functional similarity for biological processes (similarity score: 0.133) and molecular functions (similarity score: 0.221). No similarity score could be generated for cellular component as there was no significantly enriched GO term. In addition, analysis with RNAfold WebServer found that the rs3746444 polymorphism did not lead to significant effect on the folding (and thus, secondary structure formation) of the microRNA.

## Discussion

MicroRNAs are an emerging class of post-transcriptional regulators which have been implicated in oncogenesis. Polymorphisms within microRNA genes may have an impact on an individual’s susceptibility to cancer. The *MIR499A* rs3746444 polymorphism, for example, has been shown to be significantly associated with risk of cancer of the respiratory, digestive, urinary and gynecological systems^34^. Over the past decade, a growing number of studies have examined the association between *MIR499A* rs3746444 polymorphism and breast cancer susceptibility, but the results were inconsistent and often contradictory. In this work, we addressed this irregularity by pooling data from 14 previous studies which comprised 15,331 subjects (6,797 cases and 8,534 controls) via a meta-analysis. We found that the polymorphism was significantly associated with an increased breast cancer susceptibility under all five genetic models investigated.

One of the possible explanations for this observation is that the two alleles of the rs3746444 polymorphism resulted in different levels of miR-499a. In fact, it has been demonstrated previously that the variant GG genotype of the polymorphism had the lowest delta CT value (which implied a high expression level), followed by AG and AA genotypes, although it was not clear whether the difference was statistically significant^26^. It has also been reported in an *in vitro* study that miR-499a originating from the two alleles of rs3746444 polymorphism reduced the expression of tumor suppressor genes to different extents^35^. Considering these previous findings, we postulate that the variant allele of the polymorphism gave rise to a higher level of miR-499a and caused a significant decrease in the expression of tumor suppressor genes, thereby leading to an increase in breast cancer susceptibility. Besides, our *in silico* analysis showed that the A-to-G substitution in the rs3746444 polymorphism disrupted the target sites and created new target sites for a large number of genes. Genes targeted by the different alleles of the polymorphism are involved in different biological processes and have different molecular functions. We postulate that the A-to-G substitution resulted in a higher affinity of miR-499a for tumor suppressor genes, which could explain the increased breast cancer susceptibility associated with the variant allele. Nonetheless, the above postulations require further investigations as miR-499a is known to regulate not only tumor suppressor genes, but also oncogenes^9,10,12,13^.

In the present meta-analysis, a random-effect method was used in several genetic models as significant heterogeneity was observed among the studies. We addressed the heterogeneity by performing subgroup analysis according to the ethnicity of the subjects (Asians vs. Caucasians) as well as the methodological quality of the included studies. In most genetic models, subgroup analysis by ethnicity reduced the statistical heterogeneity. Interestingly, we also observed that significant association was only present in Asians, but not in Caucasians, under most genetic models. This discrepancy could be attributed to the heterogeneity in linkage disequilibrium among subjects of different ethnicities, which is a common concern in genetic association studies involving multiple populations^36,37^. Moreover, genetic association is known to be affected by gene-gene, gene-environment and gene-nutrient interactions, which might explain the disagreement in study findings between the two subgroups^38,39^. It is also worthy of mention that among the 14 studies included in the meta-analysis, only three were conducted among Caucasians. Hence, it remains a possibility that the lack of significance among Caucasians was a false negative observation due to a relatively weak statistical power^40^. Only one of the included studies was conducted among Africans; thus, subgroup analysis was not performed on Africans.

Besides, we noted that statistical heterogeneity was eliminated when only studies of high methodological quality were included in the analysis. A high quality study was defined as one which reported and fulfilled at least five of the following criteria: (i) cases were confirmed as having the malignancy, (ii) no evidence of selection bias among cases, (iii) population-based controls and genotypic distribution that conformed to HWE, (iv) appropriate control selection, (v) cases and controls were of the same ethnicity, (vi) no evidence of population stratification, (vii) genotypes validated using an independent method and investigators were blinded to the subject status, (viii) used the same genotyping method for all subjects, and (ix) genotyping call rate >99%^19^. It is unknown which of the criteria contributed to the heterogeneity. However, all low quality studies did not fulfill and/or report criteria (v), (vi), (viii) and (ix) – the former two were related to ethnic descent, which again highlighted the influence of ethnicity on the genetic association.

In this work, two formal tests were employed to assess publication bias, namely Begg’s and Egger’s tests. Both tests are commonly used in meta-analysis of genetic association studies^41^. However, there are inherent limitations associated with each test. Begg’s test does not take into account between-study heterogeneity and is more accurate when the number of included studies is large^42^. On the other hand, Egger’s test tend to give false-positive results and is more suitable for studies with continuous outcomes^43,44^. We included the two tests in our analysis to complement the limitations of each other. Both tests indicated that there was no significant publication bias in all genetic models.

There are several limitations of this meta-analysis. First, we reported only crude estimates of genetic association and did not measure gene-gene or gene-environment interactions because not all included studies contained this information. Second, the number of studies included was relatively small, especially for studies in non-Asian populations. Therefore, the association of *MIR499A* rs3746444 polymorphism with breast cancer susceptibility among non-Asian populations remained unclear. Nevertheless, a major strength of the present work is that it included a larger number of studies and subjects compared to previous reports on this topic^45,46^. Thus, the present meta-analysis provided an updated and integrated estimate of the association between the polymorphism and breast cancer risk. In addition, we performed *in silico* analysis to predict the functional impact of the polymorphism, which may help to clarify the mechanisms by which *MIR499A* rs3746444 influences breast cancer susceptibility.

In conclusion, we provided quantitative evidence that *MIR499A* rs3746444 polymorphism was associated with an elevated breast cancer susceptibility among Asians, but not among Caucasians. Additional studies is required to better clarify the clinical impact of this genetic association. Future work which takes into account gene-gene, gene-environment and gene-nutrient interactions is warranted for a more precise evidence and to further elucidate the underlying mechanism of breast cancer susceptibility.
